# The effect of long-acting dual bronchodilator therapy on exercise tolerance, dynamic hyperinflation, and dead space during constant work rate exercise in COPD

**DOI:** 10.1152/japplphysiol.00774.2020

**Published:** 2021-04-29

**Authors:** William W. Stringer, Janos Porszasz, Min Cao, Harry B. Rossiter, Shahid Siddiqui, Stephen Rennard, Richard Casaburi

**Affiliations:** ^1^The Lundquist Institute for Biomedical Innovation at Harbor-UCLA Medical Center, Torrance, California; ^2^Faculty of Biological Sciences, University of Leeds, Leeds, United Kingdom; ^3^Regeneron, Tarrytown, New York; ^4^BioPharmaceuticals R&D, AstraZeneca, Cambridge, United Kingdom; ^5^Department of Medicine, University of Nebraska Medical Center, Omaha, Nebraska

**Keywords:** COPD, CPET, exercise intolerance, hyperinflation, V_D_/V_T_

## Abstract

We investigated whether dual bronchodilator therapy (glycopyrrolate/formoterol fumarate; GFF; Bevespi Aerosphere) would increase exercise tolerance during a high-intensity constant work rate exercise test (CWRET) and the relative contributions of dead space ventilation (V_D_/V_T_) and dynamic hyperinflation (change in inspiratory capacity) to exercise limitation in chronic obstructive pulmonary disease (COPD). In all, 48 patients with COPD (62.9 ± 7.6 yrs; 33 male; GOLD spirometry stage 1/2/3/4, *n* = 2/35/11/0) performed a randomized, double blind, placebo (PL) controlled, two-period crossover, single-center trial. Gas exchange and inspiratory capacity (IC) were assessed during cycle ergometry at 80% incremental exercise peak work rate. Transcutaneous $P_{{CO}_{2}}$ (Tc$P_{{CO}_{2}}$) measurement was used for V_D_/V_T_ estimation. Baseline postalbuterol forced expiratory volume in 1 s (FEV_1_) was 1.86 ± 0.58 L (63.6% ± 13.9 predicted). GFF increased FEV_1_ by 0.18 ± 0.21 L relative to placebo (PL; *P* < 0.001). CWRET endurance time was greater after GFF vs. PL (383 ± 184 s vs. 328 ± 115 s; difference 55 ± 125 s; *P* = 0.013; confidence interval: 20–90 s), a 17% increase. IC on GFF was above placebo IC at all time points and fell less with GFF vs. PL (*P* ≤ 0.0001). Isotime tidal volume (1.54 ± 0.50 vs. 1.47 ± 0.45 L; *P* = 0.022) and ventilation (52.9 ± 19.9 vs. 51.0 ± 18.9 L/min; *P* = 0.011) were greater, and respiratory rate was unchanged (34.9 ± 9.2 vs. 35.1 ± 8.0 br/min, *P* = 0.865). Isotime V_D_/V_T_ did not differ between groups (GFF 0.28 ± 0.08 vs. PL 0.27 ± 0.09; *P* = 0.926). GFF increased exercise tolerance in patients with COPD, and the increase was accompanied by attenuated dynamic hyperinflation without altering V_D_/V_T_.

**NEW & NOTEWORTHY** This study was a randomized clinical trial (NCT03081156) that collected detailed physiology data to investigate the effect of dual bronchodilator therapy on exercise tolerance in COPD, and additionally to determine the relative contributions of changes in dead space ventilation (V_D_/V_T_) and dynamic hyperinflation to alterations in exercise limitation. We utilized a unique noninvasive method to assess V_D_/V_T_ (transcutaneous carbon dioxide, Tc$P_{{CO}_{2}}$) and found that dual bronchodilators yielded a moderate improvement in exercise tolerance. Importantly, attenuation of dynamic hyperinflation rather than change in dead space ventilation was the most important contributor to exercise tolerance improvement.

## INTRODUCTION

Patients with chronic obstructive pulmonary disease (COPD) experience expiratory flow limitation, breathlessness, dyspnea, and reduced exercise tolerance relative to age and sex matched controls (1–4). A prominent mechanism responsible for exercise intolerance in COPD is thought to be dynamic hyperinflation (DH) during exercise due to end expiratory lung volume increase, usually assessed by a commensurate decrease in inspiratory capacity (IC) (5, 6). The effects of dynamic hyperinflation on dyspnea and exercise intolerance are amplified in COPD by an increased ventilatory requirement for exercise, related to increased dead space to tidal volume ratio (V_D_/V_T_) (7, 8). Treatment with bronchodilators (BD) are partially effective at reducing expiratory flow limitation, and the optimal regimen in COPD appears to be achieved with a fixed dose, long-acting inhaled combination medications (LABA/LAMA, long-acting beta agonists and long-acting muscarinic antagonists) (1, 2, 9–11). In patients with COPD, LABA/LAMA bronchodilator therapy reduces DH and increases exercise tolerance (12). However, the effect of LABA/LAMA bronchodilator therapy on V_D_/V_T_ during exercise in COPD is controversial (13). Although short-acting bronchodilator therapy in COPD does not influence estimated V_D_/V_T_, long-acting bronchodilation, which increases ventilation to both well- and poorly perfused lung units, has the potential to worsen V_D_/V_T_ and increase ventilatory requirement. LABA/LAMA bronchodilators also increase pulmonary blood flow, as demonstrated using gadolinium-enhanced magnetic resonance imaging at rest (14). Therefore, should the negative effects of COPD on alveolar ventilation to pulmonary blood flow ratio (V̇_A_/Q̇) distribution during exercise be ameliorated by LABA/LAMA bronchodilators, then V_D_/V_T_ may be reduced and, thus, contribute to the mechanism by which LABA/LAMA bronchodilators increase exercise tolerance.

We hypothesized that glycopyrrolate/formoterol fumarate (GFF, Bevespi Aerosphere) would increase exercise tolerance during high intensity, constant work rate, cycle ergometer exercise in stable COPD patients by simultaneously reducing DH and V_D_/V_T_ during exercise. To assess the relative contributions of these two variables, we aimed to determine DH using serial IC measurements and breath-by-breath V_D_/V_T_ using transcutaneous $P_{{CO}_{2}}$ (Tc$P_{{CO}_{2}}$) measurements and compare these measurements at isotime during constant work rate exercise to intolerance while receiving GFF or placebo (PL).

## METHODS

The study was approved by the Institutional Review Board of The Lundquist Institute for Biomedical Innovation at Harbor-UCLA Medical Center (Study No. 21752-01). Written informed consent was obtained from each subject before participation.

### Study Design

This was a single center, randomized, double blind, PL controlled, two-period, crossover clinical trial conducted between March 2017 and February 2019 (NCT03081156).

### Participants

Male or females were enrolled, who were between 40 and 80 yr with a clinical diagnosis of COPD (postalbuterol FEV_1_/FVC ratio <0.70) and stable, without change in medications or exacerbation within the prior 4 wk. Participants were current or ex-smokers with >10 pack-years smoking history. Exclusion criteria included: significant diseases other than COPD that affect exercise tolerance: e.g., a history of heart failure or arthritis; a documented history of childhood or family asthma, treatment with oral corticosteroid medications above 10 mg/day, daytime oxygen use >6 hours/day, desaturation (from pulse oximetry) during exercise to <80%; a physical contraindication for exercise, e.g., marked exercise induced hypertension, serious arrhythmia; and other unstable conditions as evaluated by the principal investigator. Participants were categorized into Global Initiative for Chronic Obstructive Lung Disease (GOLD) spirometry groups: GOLD 1 (FEV_1_ ≥ 80% predicted), GOLD 2 (50% ≤ FEV_1_ predicted >80%), GOLD 3 (30% ≤ FEV_1_ predicted >50%), GOLD 4 (FEV_1_ < 30% predicted).

### Procedures

#### Pulmonary function testing.

Participants continued their usual medications for screening. Prebronchodilator spirometry, body plethysmography, and single-breath diffusing capacity of the lung for carbon monoxide (DL_CO_) were measured according to recommended American Thoracic Society/European Respiratory Society (ATS/ERS) standards (15–18). Postbronchodilator spirometry was measured 20 min after inhalation of 4 puffs of albuterol (400 mg). Predicted values for spirometry and vital capacity (VC) were from National Health and Nutrition Examination Survey (NHANES) III (19); predicted lung volumes were from ERS (20); and predicted DL_CO_ was from Cotes et al. (21).

#### Chest computed tomography.

Subjects underwent a volumetric computed tomography (CT) scan of the chest at full inspiration and relaxed expiration. For this study, inspiratory CT data were used. Emphysema was defined as percentage of lung attenuation areas below −950 Hounsfield Units. Airway wall thickening was measured using mean segmental wall thickness in mm of a theoretical airway of 10-mm luminal perimeter (22, 23).

#### Cardiopulmonary exercise testing.

Symptom-limited exercise tests were performed using cardiopulmonary measurements of gas exchange and ventilatory variables (Vmax Encore, Vyaire, Yorba Linda, CA, or CPX Ultima, Medical Graphics Corporation, St. Paul, MN) with electromagnetically braked cycle ergometry (Excalibur Sport PFM, Lode, Groningen, NL). Each subject started and finished all exercise tests (incremental and constant work rate) using the same cardiopulmonary system and cycle ergometer. Daily calibration as well as monthly biological calibrations were performed according to vendor recommendations and guidelines (24).

#### Incremental exercise testing.

Participants were screened with a ramp-incremental exercise test at 10 Watts (W)/min. A 3-min rest period was followed by 3 min of unloaded cycling, then increasing work rate (10 W/min) until intolerance, determined as the inability to maintain pedaling cadence >50 rpm despite encouragement. The test was concluded with at least 3 min of unloaded cycling. Subjects breathed through a mouthpiece with a nose clip in place. Heart rate was derived from a 12-lead electrocardiogram. Inspiratory capacity (IC) was measured every 2 min according to ATS standards (25, 26). Blood pressure (sphygmomanometer) and ratings of breathlessness and leg effort (modified Borg CR-10 scale) were also assessed every 2 min. Maximal voluntary ventilation (MVV) was estimated as FEV_1_ × 40, where FEV_1_ was measured seated on the cycle ergometer immediately before exercise testing (26). Peak work rate was determined as the value during the last 10 s of exercise. For variables measured breath-by-breath, peak values were defined as the average of the last 30 s of exercise averaged over three 10-s time bins. For variables assessed every 2 min, end-exercise values were taken as the last value during exercise before or during that 30-s window. The estimated lactate threshold (LAT) was assessed using the V-slope method and corroborated with ventilatory equivalent and end-tidal partial pressure responses ($\text{P}\text{E}{\text{T}}_{{\text{O}}_{2}}$and $\text{P}\text{E}{\text{T}}_{\text{C}{\text{O}}_{2}}$) (27). For V_E_/$V_{{CO}_{2}}$ slope, data calculated as 30-s averages were fit by linear regression. The lower point for the regression was at a work rate above 20 watts and the upper point was at the respiratory compensation point. Predicted values for ramp exercise variables were those of Hansen et al. (26).

#### Constant work rate exercise testing.

The work rate for constant work rate exercise testing (CWRET) was 80% of the peak work rate measured during incremental exercise. A 3-min rest period was followed by 3 min unloaded cycling, then the work rate was increased as a step change to 80% peak incremental work rate. Exercise tolerance during CWRET was determined at the time between the start of loaded exercise (work > 0 watts) and when the subject could no longer maintain pedaling cadence >50 rpm despite encouragement (specifically, the time between the 10-s bin in which loaded cycling commenced and the 10-s window in which the work rate decreased). The test was concluded with at least 3 min of unloaded cycling. CWRET was initially performed at screening. If exercise duration was outside the 3–8 min guideline duration (4), a second screening CWRET was performed at an ∼5 W higher or lower work rate—whichever was appropriate to obtain a 3–8-min CWRET duration (28). In analysis of these data, for variables measured breath-by-breath, end-exercise values were defined as the average of the last 30 s of exercise averaged over three 10-s time bins. For variables assessed every 2 min, end-exercise values were taken as the last value during exercise before or during that 30-s window. For physiologic variables, isotime responses were taken as the time of the last 10-s time bin in the shorter of the study drug and placebo tests. For variables assessed every 2 min, isotime values were taken as the value recorded at or immediately preceding isotime.

#### V_D_/V_T_ estimation.

Transcutaneous CO_2_ (Tc$P_{{CO}_{2}}$) and oxygen saturation by pulse oximetry ($Sp_{O_{2}}$; Tosca 500, Radiometer America, Brea, CA) was recorded breath-by-breath during incremental and constant work rate exercise. The sensor was attached to the left earlobe ∼10 min before exercise testing to allow the Tc$P_{{CO}_{2}}$ measurement to stabilize. The device was set to a probe temperature of 44°C. Tc$P_{{CO}_{2}}$ was substituted for $\text{P}{\text{a}}_{\text{CO}}{}_{{}_{2}}$ in Eq. *1* for dead space estimation, and the fraction of the dead space of resulting from the mouthpiece breathing apparatus (V_DS_ = 110 mL) was subtracted from the total.

(*1*) $$V_{D}/V_{T}=\;\left(1-\left[\text{863}/\left(TcP_{{CO}_{2}}*˙\text{V}\text{E}/V_{{CO}_{2}}\right)\right]\right)-\left(V_{\text{DS}}/V_{T}\right)$$

### Protocol

**Figure 1.**
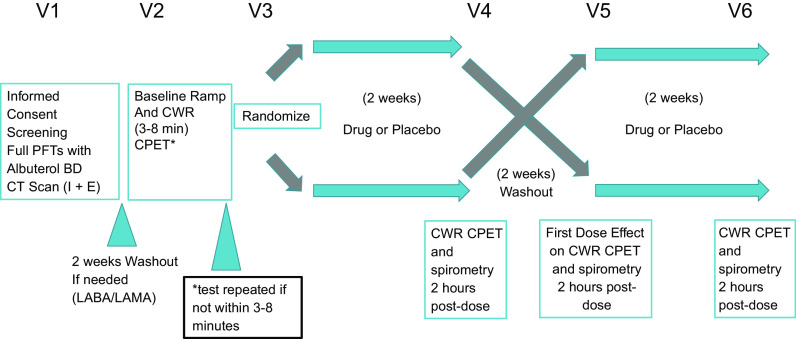
Protocol and visit designation for this single-center, randomized, double-blind, placebo-controlled, two-period, crossover clinical trial. CPET, cardiopulmonary exercise testing; CT, computed tomography; CWR, constant work rate; LABA, long-acting beta agonists; LAMA, long-acting muscarinic antagonists; PFT, pulmonary function testing; V, visit.

The study protocol diagram is provided in Fig. 1. *Visit 1* included obtaining informed consent, vital signs including $Sp_{O_{2}}$; a focused physical examination; full pulmonary function testing (PFT) on usual respiratory medications; 6-min walk test; BODE (Body Mass index, Airflow Obstruction, Dyspnea, Exercise Capacity) index (29); modified Medical Research Council (mMRC) dyspnea score (30); inspiratory and expiratory (I + E) CT scan (31); and assessment of exacerbations in the past year (defined as new antibiotic prescription, new or increased oral corticosteroids, or a visit to the emergency room or hospital).

Eligible participants underwent a 2-wk washout period where all LABA and LAMA bronchodilators were withheld. Short-acting beta agonists (SABA) and/or short-acting muscarinic antagonists (SAMA) were allowed during washout. Participants who were currently using inhaled corticosteroids before enrollment (either individual or as a component of a combined inhaler) were continued on a single inhaled corticosteroid agent at appropriate dosages (beclomethasone dipropionate; QVAR). Participants who were not using LABA or LAMA therapy did not require washout.

After the appropriate washout period, participants performed screening ramp-incremental and CWRET exercise tests (*visit 2*) separated by at least 1 h of rest. Participants and study personnel were blinded to the statistician created randomization that the research pharmacist followed to provide either PL first or GFF for *visits 3* and *5*. A coded, indistinguishable inhaler (either GFF or PL) was dispensed at *visit 3* and used for 2 wk. At *visits 4* and *6* spirometry was followed by CWRET. *Visit 5* was preceded by another 2-wk washout period, after which participants received their crossed-over inhaler (either PL or GFF) and took the first dose in the clinic. The acute effect of either PL or GFF on spirometry was determined 2 h postdose on *visit 5*. Participants then resumed their prestudy medications, and a follow-up visit (*visit 7*) was performed 1 wk later to assess patient safety.

### Statistics

Data are presented as mean ± SD or 95% confidence interval (CI). Differences in physiologic and perceptual responses between study drug and placebo were analyzed using paired Student’s *t* tests or repeated measures analysis of variance (rmANOVA). Statistical significance was accepted at *P* < 0.05. Multivariable linear regression analysis was used to determine whether differences between study drug and placebo for a variety of physiologic variables were predictive of increases in endurance time (Sigmaplot v.14, Systat Software, San Jose, CA). The sample size was estimated assuming an effect size on endurance time of 60 s [lower bound of the minimum clinically important difference (MCID) estimated for CWRET (32)] and a standard deviation of change in endurance time of 150 s (4). A desired power (1 − β) of 0.8 and α of 0.05 resulted in a sample size of 50 participants.

## RESULTS

### Participants

Sixty participants were consented. Eight subjects failed screening procedures related to failure to meet pulmonary function inclusion criteria (5), recent hospitalization for heart failure (1), chest mass found on the study CT scan (1), and inability to tolerate bronchodilator washout (1). The four subjects who entered, but did not complete the study, all withdrew consent in the prerandomization period (before *visit 3*), related to family emergency (1), knee pain (1), and unhappiness with the possibility of being in the placebo group (1); one subject declined to give a reason for withdrawal. Forty-eight subjects completed the study.

Participant demographics were: age 62.9 ± 7.6 yr; height 172.5 ± 8.6 cm; weight 86.7 ± 18.4 kg; body mass index 29.1 ± 5.9 kg/m^2^; gender: 33 male (69%); race: 26 Caucasian (54%); 21 African American (43%); 1 Asian (2%). GOLD spirometry stage 1/2/3/4 was *n* = 2/35/11/0. Thirty were past smokers, 18 were current smokers. The average pack-years of cigarette smoking was 38 ± 21. All participants were stable for at least 4 weeks before screening, but 11 (23%) had experienced a COPD exacerbation within the prior year and one subject had experienced two exacerbations. At screening, the mean 6-min walk distance was 393 ± 86 m, BODE (8) index was 1.83 ± 1.36 and mMRC score was 1.75 ± 0.5. Five (11%) subjects had been receiving LABA, four (8%) had been receiving LAMA therapy; nine (19%) had been receiving dual LABA/LAMA therapy. Nineteen participants (40%) had been receiving inhaled corticosteroids, which were continued during the study using inhaled beclomethasone at a dosage of either 40 or 80 µg depending on the previous dosage. None of the subjects had been receiving PDE4 inhibitors or daily azithromycin. Average hemoglobin concentration was within the normal range (14.4 ± 1.6 g/dL) and the average eosinophil count was 209 ± 165 cells/uL or 2.66 ± 1.79%. CT scans (*n* = 46, 2 subjects had recent CT scans, which were not repeated for the study) demonstrated emphysema 9.09 ± 7.71% and mean segmental wall thickness 3.61 ± 0.47 mm.

### Pulmonary Function

Screening pulmonary function with usual medications and before washout are presented in Table 1. The majority of participants had mild-moderate (71%) or severe (23%) COPD. The FEV_1_/FVC ratio was 51.8 ± 11.6%. After 4 puffs of albuterol, the average FEV_1_ increase (0.14 ± 0.14 L and 9.1 ± 9.4%) and FVC increase (0.21 ± 0.30 L and 6.2 ± 8.8%) did not fulfill ATS/ERS criteria for a positive bronchodilator response (17). The subjects on average were not hyperinflated with respect to TLC (101 ± 17% predicted); however, the RV (118 ± 45%) and FRC (110.5 ± 39.9%) were elevated. The average gas transfer/DL_CO_ was reduced at 59.3 ± 16.2% of predicted with DL_CO_ ranging from 23 to 88% of predicted.

**Table 1.** Baseline pre- and postbronchodilator pulmonary function (n = 48)

	Prebronchodilator	Postbronchodilator (Albuterol)	Bronchodilator Effect (Albuterol)	% Increase
FEV_1_, L	1.72 ± 0.56	1.86 ± 0.58	0.14 ± 0.14	9.1 ± 9.4%
FEV_1_, % Predicted (19)	58.6 ± 13.8	63.6 ± 13.9	-	-
FVC, L	3.35 ± 0.89	3.56 ± 0.98	0.21 ± 0.30	6.2 ± 8.8%
FVC, % Predicted (19)	87.1 ± 14.4	92.2 ± 15.3	-	-
FEV_1_/FVC, % (19)	51.8 ± 11.6	53.2 ± 11.4	-	-
TLC, L	6.15 ± 1.72	-	-	-
TLC, % Predicted (20)	101 ± 17	-	-	-
FRC, L	3.55 ± 1.57	-	-	-
FRC, % Predicted	110.5 ± 39.9	-	-	-
RV, L	2.68 ± 1.19	-	-	-
RV, % Predicted	118 ± 45	-	-	-
RV/TLC, %	42.8 ± 8.9	-	-	-
IC	2.60 ± 0.66	-	-	-
IC, % Predicted	98 ± 22	-	-	-
DL_CO_, mL/min/mmHg	15.2 ± 4.9	-	-	-
DL_CO_, % Predicted (21)	59.3 ± 16.1	-	-	-
CT emphysema, % < LA 950*	9.09 ± 7.71	-	-	-
CT mean segmental wall thickness, mm*	3.61 ± 0.47	-	-	-

All values, mean ± SD. FEV_1_, forced expiratory volume in 1 s; FVC, forced vital capacity; TLC, total lung capacity; RV, residual volume; DL_CO_, diffusing capacity of the lung for carbon monoxide. *CT, computed tomography, *n* = 46 (2 subjects had a recent CT and did not repeat a quantitative CT for the study).

At *visit 5*, 23 subjects received GFF and 25 subjects received PL per their randomization sequence. Spirometry obtained 2-h after GFF or placebo inhalation at *visit 5* following a 2-wk wash out from long-acting bronchodilators (LABA/LAMA) is displayed in Table 2. After administration of GFF, FEV_1_ increased by 0.18 ± 0.20 L (11.8 ± 15.6%, *P* < 0.001 versus predose). There was no increase in FEV_1_ following PL (0.01 ± 0.10 L; *P* > 0.050 vs. predose).

**Table 2.** The effect of first dose of LABA/LAMA bronchodilator (GFF) or placebo (PL) on spirometry at visit 5

	Subjects Receiving Placebo (*n* = 25)		Subjects Receiving GFF (*n* = 23)	
	**Predose**	**Postdose Change**	**Predose**	**Postdose Change**
FEV_1_, L	1.79 ± 0.64	0.01 ± 0.10	1.64 ± 0.55	0.18 ± 0.21#
FVC, L	3.62 ± 1.14	0.03 ± 0.26	3.21 ± 0.82	0.17 ± 0.23*

Postdose change is the difference in the variables (FEV_1_ and FVC) between predose and 120 min postdose of either PL or GFF at *visit 5*. FEV_1_, forced expiratory volume in 1 s; FVC, forced vital capacity; GFF, glycopyrrolate/formoterol fumarate; LABA, long-acting beta agonists; LAMA, long-acting muscarinic antagonists.

*P ≤ 0.05, #*P* ≤ 0.001, Student’s *t* test.

### Incremental Exercise Test

Table 3 presents the baseline characteristics during screening ramp-incremental exercise. Participants had impaired aerobic function: reduced V̇o_2_ peak [1.36 L/min, 67 ± 19% predicted (26), 15.8 ± 4.9 mL/kg/min], low LAT (0.88 ± 0.20 L/min, 92% ± 21 of predicted lower limit of normal). They had a peak work rate of 91 ± 33 W, abnormal indices of ventilatory efficiency and gas exchange, including a high V̇_E_/MVV (0.71 ± 0.16), increased V̇_E_/V̇co_2_ at the LAT (34.5 ± 5.1), and V̇_E_ versus V̇co_2_ slope (29.5 ± 6.3). Twenty-two of the 48 subjects (46%) had a peak V̇_E_/MVV above 0.75, and subjects showed signs of dynamic hyperinflation, as evidenced by a fall in IC, with inspiratory reserve volume (IRV, IC-V_T_) averaging 0.60 ± 0.40L at end exercise. The average V_D_/V_T_ for the group at the LAT was 0.29 ± 0.08.

**Table 3.** Baseline ramp incremental exercise testing results (n = 48)

Incremental Exercise Test	Means ± SD
Peak V̇o_2_, L/min	1.36 ± 0.41
Peak V̇o_2_, mL/kg/min	15.8 ± 4.9
Peak V̇o_2_, % Predicted	67 ± 19
Peak Work Rate, W	91 ± 33
V̇o_2_ at LAT, L/min	0.88 ± 0.20
V̇o_2_ at LAT, % Predicted	92 ± 21
Work rate at LAT, W	44.3 ± 15.8
Peak HR, beat/min	123 ± 17
O_2_ saturation at peak exercise, %	97 ± 3
Peak O_2_ pulse, mL/beat	11.0 ± 2.8
Peak V̇_E_, L/min	52.3 ± 20.2
Peak V̇_E_/V̇co_2_	37.0 ± 7.0
V̇_E_/V̇co_2_ at LAT	34.5 ± 5.1
V̇_E_ vs. V̇co_2_ slope	29.5 ± 6.3
V_D_/V_T_ at the LAT	0.29 ± 0.08
Peak V̇_E_/MVV	0.71 ± 0.16
Peak V̇_E_/MVV above 0.75 [*n* (%)]	22 (18)
ΔIC (peak-rest), L	−0.41 ± 0.46
Peak IRV, *L*	0.60 ± 0.40

V̇o_2_, oxygen uptake; LAT, estimated lactic acidosis threshold; for V̇o_2_ at LAT the % predicted is 40% of peak V̇o_2_; V̇_E_, minute ventilation; V̇co_2_, carbon dioxide output; V̇_E_ vs. V̇co_2_ slope, the slope of the relationship between V̇_E_ and V̇co_2_ measured between 20 W and the respiratory compensation point; WR, work rate (watts); MVV, maximum voluntary ventilation estimated from forced expiratory volume in 1 s × 40; ΔIC (peak-rest), the difference in inspiratory capacity between resting and peak exercise; IRV, inspiratory reserve volume. Peak HR, maximum HR recorded during the cardiopulmonary exercise testing. O_2_ saturation at peak exercise. Peak O_2_ pulse: peak V̇o_2_/peak HR. All predicted values from Wasserman et al. (26).

### Constant Work Rate Exercise Test

**Figure 2.**
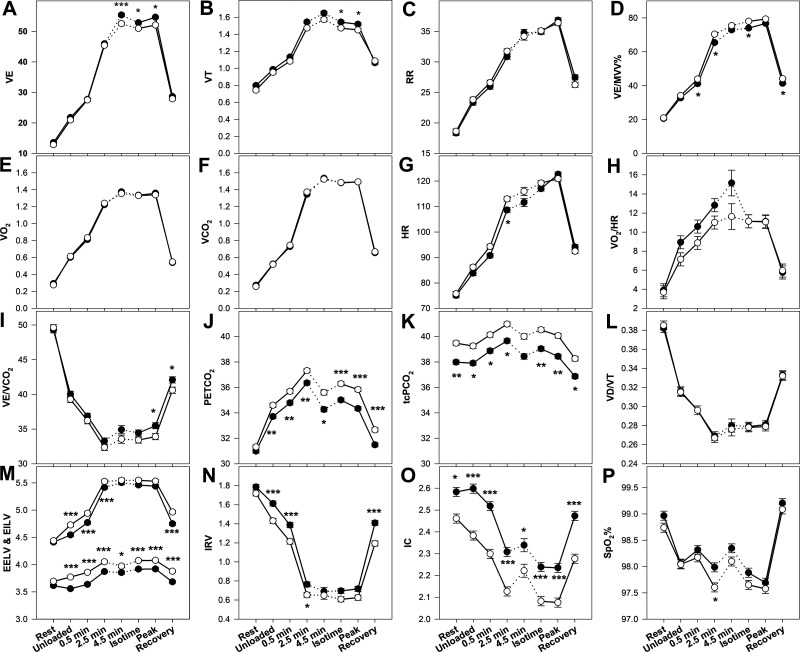
*A–P*: physiologic responses to constant work rate exercise testing in patients with COPD receiving LAMA/LAMA therapy (closed circles) vs. Placebo (open circles). Description of visits is given in text. All data points are for 48 subjects, except the 4.5 minute time point, in which only 22 subjects completed this duration in both tests. *P < 0.05, **P = 0.01, ***P ≤ 0.001 by rmANOVA. V̇_E_ (L/min), pulmonary ventilation; V_T_ (L), tidal volume; RR (breaths/min), respiratory rate; V̇_E_/MVV (%), pulmonary ventilation divided by maximum voluntary ventilation; V̇o_2_ (L/min), oxygen uptake; V̇co_2_ (L/min), carbon dioxide output; HR (beats/min), heart rate; V̇o_2_/HR (mL/beat), oxygen pulse; V̇E/V̇co_2_, pulmonary ventilation divided by carbon dioxide output; $PET_{CO_{2}}$ (mmHg), end-tidal carbon dioxide partial pressure; Tc$P_{{CO}_{2}}$ (mmHg), transcutaneous carbon dioxide partial pressure; V_D_/V_T_, dead space to tidal volume ratio; IRV (L), inspiratory reserve volume; IC (L), inspiratory capacity; $Sp_{O_{2}}$ (%), oxygen saturation by pulse oximetry. COPD, chronic obstructive pulmonary disease; LAMA, long-acting muscarinic antagonists.

**Figure 3.**
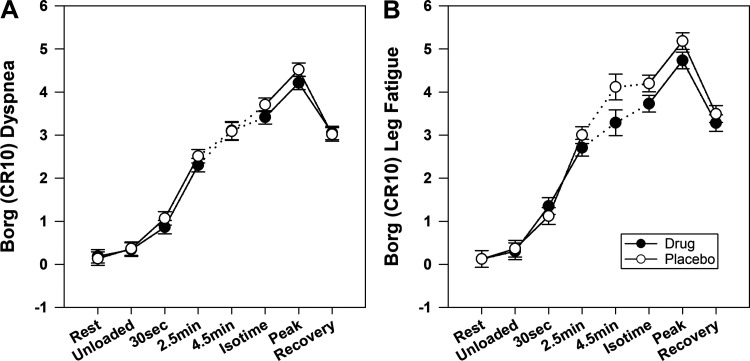
Perceptual responses to constant work rate exercise in patients with COPD receiving LAMA/LAMA therapy (closed circles) vs. Placebo (open circles). All data points are for 48 subjects except the 4.5-min time point, in which only 22 subjects completed this duration in both tests. There were no statistical differences in the Borg scores for either dyspnea (*A*) or leg fatigue (*B*). COPD, chronic obstructive pulmonary disease; CR10, Category and Ratio Scale of 10; LAMA, long-acting muscarinic antagonists.

**Figure 4.**
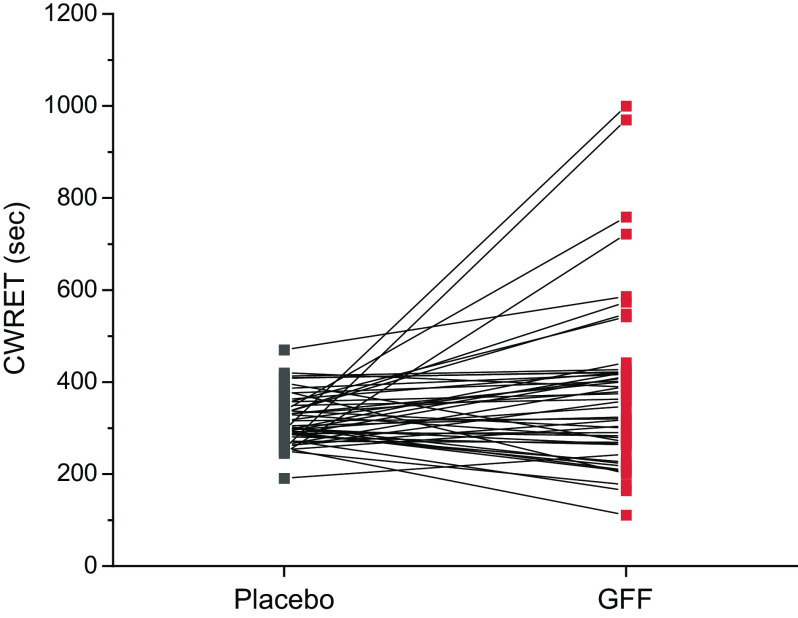
Constant work rate exercise duration while receiving dual bronchodilator vs. placebo for 48 individual subjects with COPD. COPD, chronic obstructive pulmonary disease; CWRET, constant work rate exercise time; placebo versus GFF, dual LABA/LAMA bronchodilator; GFF, glycopyrrolate/formoterol fumarate; LABA, long-acting beta agonists; LAMA, long-acting muscarinic antagonists.

Figures 2 and 3 present the time course of a number of physiologic and perceptual responses to CWR exercise in response to GFF and placebo. The average CWRET work rate was 73 ± 27 W. The endurance time (primary outcome) was 383 ± 184 s on GFF versus 328 ± 115 s on PL, with a mean individual difference of 55 ± 125 s (17% increase; *P* = 0.013, CI 20–90 s). Figure 4 displays the individual CWRET responses in seconds for 48 subjects on placebo and GFF. There were large increases in exercise tolerance in certain individuals, with the average increasing by 55 ± 125 s. The MCID for endurance time for CWRET has been postulated to be 100 s or 33% (34); by both of these metrics, 18 of 48 subjects, demonstrated clinically important increases in exercise tolerance. Isotime occurred 4.53 ± 1.45 min after the onset of loaded CWRET exercise.

In Fig. 2*A*, V̇_E_ increased progressively during CWRET in both GFF (solid circles) and Placebo (open circles) studies, but were little changed from 2.5 min onward. V̇_E_ was statistically significantly higher with GFF versus Placebo at 4.5 min, isotime, and peak exercise time points. In Fig. 2*B*, tidal volume (V_T_) peaked at approximately ∼ 1.5–1.6 L and decreased thereafter; however, V_T_ in GFF remained statistically significantly higher than placebo at isotime and peak exercise. In Fig. 2*C*, respiratory rate increased progressively throughout exercise with an acceleration at peak exercise, but the time course was essentially identical in GGF and placebo studies. In Fig. 2*D*, V̇_E_/MVV was slightly lower throughout exercise in the GFF studies; this achieved statistical significant at 30 s, 2.5 min, isotime, and recovery.

In Fig. 2, *E* and *F*, both V̇o_2_ and V̇co_2_ were unchanging from 4.5 min through isotime and peak exercise at ∼1.35 and 1.50 L/min, respectively, with no significant differences between the GFF versusplacebo studies. In Fig. 2*G*, HR progressively increased throughout exercise, with values slightly lower in GFF than placebo studies during exercise, but only the 2.5 min difference achieved statistical significance. In Fig. 2*H*, O_2_ pulse (V̇o_2_/HR) rose during exercise, but no differences between GFF and placebo achieved statistical significance.

In Fig. 2*I*, V̇_E_/V̇co_2_ was higher in the GFF group relative to placebo throughout exercise; however, this difference achieved statistical significance at peak exercise and recovery. In Fig. 2*J*, the end tidal CO_2_ values were statistically significantly lower in GFF versus placebo (by ∼1 mmHg) throughout exercise and recovery. In Fig. 2*K*, transcutaneous $P_{{CO}_{2}}$ (Tc$P_{{CO}_{2}}$) was statistically significantly lower at rest, during all levels of exercise, and recovery relative to placebo also by ∼ 1 mmHg. In Fig. 2*L*, V_D_/V_T_ was essentially identical at all points of the study with no statistically significant differences.

In Fig. 2*M*, end-inspiratory lung volume was lower with GFF at 30 s, 2.5 min, and 4.5 min relative to placebo, but was not statistically different at isotime or peak exercise. For end-expiratory lung volume, GFF was statistically significantly lower at all times during exercise as well as in recovery compared to placebo. In Fig. 2*N*, IRV with GFF remained slightly higher than placebo throughout exercise, and was statistically different at unloaded cycling, 30 s, 2.5 min, and recovery. In Fig. 2*O*, IC remained statistically significantly higher in GFF as compared to placebo studies throughout all measured points of rest, exercise, and recovery. In Fig. 2*P*, the oxygen saturation was not statistically different between GFF and placebo except at the 2.5 min time point where saturation was lower in the placebo group.

Figure 3*A* presents the Borg Category and Ratio Scale of 10 (CR10) scores for dyspnea during rest, exercise, and recovery. GFF trended lower versusplacebo, but no significant differences were observed. In Fig. 3*B*, the Borg CR 10 scores for leg fatigue demonstrated trends for lower scores on GFF relative to placebo, but no significant differences emerged.

The increase in isotime IC was not significantly correlated with the endurance time improvement with BFF. For the 46 subjects who had quantitative CT scans, percent emphysema and segmental wall thickness were not significantly correlated with constant work rate endurance time improvement (*R*^2^ = 0.0043 and 0.066, respectively). There was also no correlation of V_D_/V_T_ change with these CT parameters. DL_CO_ as % predicted also showed no significant correlation with endurance time improvement (*R*^2^ = 0.0086).

## DISCUSSION

The physiologic mechanisms limiting exercise in COPD include increased ventilatory requirements consequent to one or more mechanisms including, greater V̇_A_/Q̇ inequality, lower LAT, larger V_D_/V_T_, and abnormal ventilatory mechanics (dynamic hyperinflation; constrained V_T_; lower IRV; increased respiratory muscle work) (3, 7, 8, 26, 35, 36). These mechanisms are not exclusive, and may potentiate one another, exacerbating dyspnea and reducing exercise tolerance. This study aimed to determine the contributions of alterations in DH and V_D_/V_T_ to the increases in exercise tolerance observed with LABA/LAMA bronchodilator therapy in COPD. We found, as anticipated, that LABA/LAMA bronchodilator therapy (GFF) significantly increased exercise tolerance in patients with mild to very severe COPD (∼17%), while reducing isotime dynamic hyperinflation (on average by ∼150 mL or ∼ 7% increase in IC) without significant isotime change in V_D_/V_T_. Together these data suggest that the primary mechanism by which LABA/LAMA bronchodilator therapy increases exercise tolerance in patients with COPD is via beneficial effects on respiratory mechanics to reduce dynamic hyperinflation, rather than increased ventilatory efficiency (reduced V_D_/V_T_).

The increase in exercise tolerance LABA/LAMA treatment in this placebo controlled, double blinded, study was modest (17%). The average increase in exercise tolerance by 55 s (CI 20–90 s) was less than the consensus MCID of 105 s or 33% increase, based on interventions including bronchodilator therapy, pulmonary rehabilitation, oxygen supplementation and noninvasive ventilation (4). Our findings were, however, approximately equal to the lower 95% confidence interval of the MCID (60 s or 22% increase) (4) and similar to previous studies of LABA/LAMA or short-acting beta agonists (SABA) and short-acting muscarinic antagonists (SAMA) therapy (4). A subanalysis of our data showed that the average increase in exercise tolerance tended to be greater in GOLD 3–4 than GOLD 1–2 participants (124 ± 143 s, or 38% increase vs. 34 ± 144 s or 10% increase; *P* = 0.076). Although our study was not powered to identify the effect of combination LABA/LAMA across disease severities, these observations are consistent with earlier studies that found that bronchodilator therapy is less effective in patients with COPD with mild obstruction in whom exercise limitation may be dominated by peripheral muscle function (2).

Assessment of ventilatory inefficiency during exercise is challenging. The ratio of ventilation to CO_2_ output (V̇_E_/V̇co_2_) at LAT (or nadir) is commonly used to infer inefficient ventilation. However, because V̇_E_/V̇co_2_ depends on both ventilatory efficiency (V_D_/V_T_) and $\text{P}{\text{a}}_{\text{CO}}{}_{{}_{2}}$, it is inaccurate to determine the normalcy (or not) of V_D_/V_T_ in patients with intermediate V̇_E_/V̇co_2_ values (32). Further, using end-tidal $P_{{CO}_{2}}$ values (rather than arterial $P_{{CO}_{2}}$) to calculate V_D_/V_T_ is highly inaccurate, in all but normal subjects (37–39). The most appropriate method to calculate V_D_/V_T_ uses $\text{P}{\text{a}}_{\text{CO}}{}_{{}_{2}}$, utilizing the modified Bohr-Enghoff correction (40, 41). However, the blood draws during exercise that this necessitates, either from an indwelling catheter or from a single arterial puncture, is painful, time consuming and yields poor time resolution. $\text{P}{\text{a}}_{\text{CO}}{}_{{}_{2}}$ may be estimated noninvasively using transcutaneous $P_{{CO}_{2}}$ (33, 34, 42–45), and may be reasonably applied (i.e., have good agreement with $\text{P}{\text{a}}_{\text{CO}}{}_{{}_{2}}$) to estimate V_D_/V_T_ on a breath-by-breath basis during a cardiopulmonary exercise testing in patients with COPD (37).

To address the mechanism(s) of the observed improvement in exercise tolerance resulting from LABA/LAMA therapy, we assessed both dynamic hyperinflation using serial inspiratory capacity maneuvers every 2 min and the V_D_/V_T_ calculated breath-by-breath using Tc$P_{{CO}_{2}}$. At isotime during constant work rate exercise, we observed that LABA/LAMA bronchodilator therapy yielded significantly greater tidal volume, allowing greater ventilation and V̇_E_/V̇co_2_ for an identical exercise task (Fig. 2*B*). End-exercise comparisons also showed greater V̇_E_, V̇_E_/V̇co_2_, and V_T_ as well as higher Tc$P_{{CO}_{2}}$ with bronchodilator therapy, consistent with relief of pulmonary mechanical constraints (Fig. 2, *A*, *B*, *I*, and *K*). Average respiratory rate was not statistically different. However, the overall effect on V_D_/V_T_ both at isotime and at end-exercise for GFF versusplacebo was negligible, and did not reach statistical significance. Therefore, contrary to our hypothesis, increased exercise tolerance following LABA/LAMA therapy in COPD was not associated with reduced exercising V_D_/V_T_. Consistent with the literature, increased exercise tolerance following LABA/LAMA therapy in COPD was coincident with an attenuated fall in IC (reduced dynamic hyperinflation).

Some previous studies (but not all) suggest that V_D_/V_T_ is increased following bronchodilation therapy (14, 46, 47). Elbehairy et al. studied 20 patients with COPD who performed constant work rate exercise following acute administration of LABA/LAMA bronchodilators versus placebo; no difference in isotime V_D_/V_T_ was detected (7). Marvin et al. (47) studied 15 men with COPD who performed 6 min of low-intensity constant work rate exercise after receiving oral theophylline plus an oral short acting beta agonist or placebo for 10 days; a trend for a greater exercise-induced fall in V_D_/V_T_ was seen with the combination oral bronchodilator (23). Vogel-Claussen et al. utilized gadolinium-enhanced MRI and phase-resolved functional lung MRI to measure pulmonary microvascular blood flow and regional ventilation, respectively, in 62 patients with COPD receiving 2 wk of LABA/LAMA versus placebo; though V_D_/V_T_ was not directly measured, LABA/LAMA therapy was found to decrease both nonventilated and hypoventilated lung regions (14).

This could occur by increased ventilation of poorly perfusion lung regions following bronchodilation in the proximal airways. Nevertheless, our RCT found no difference between V_D_/V_T_ at isotime in GFF and PL groups (Fig. 2*L*), suggesting that GFF does not have a negative effect on V̇_A_/Q̇ inequality during exercise in COPD, leading to an increase in V_D_/V_T._

Although this was a rigorous physiologic study of a pharmaceutical intervention in COPD subjects, there were some weaknesses. First, the small overall effect size on endurance time was likely due to the majority of participants (75%) being mild or moderately obstructed. The effects on exercise tolerance and V_D_/V_T_ may have been different if more severe patients had been included in the study. Second, V_D_/V_T_ estimation relied on Tc$P_{{CO}_{2}}$ measurements and we did not draw simultaneous arterial bloods samples for $\text{P}{\text{a}}_{\text{CO}}{}_{{}_{2}}$.

We conclude that combination bronchochodilator therapy (glycopyrrolate/formoterol fumarate; GFF) increases exercise tolerance in patients with COPD by attenuating dynamic hyperinflation without increasing dead space ventilation (V_D_/V_T_).

## GRANTS

AstraZeneca provided funding for this project through an investigator-initiated grant provided to the Lundquist Institute for which Dr. W. W. Stringer was the principal investigator.

## DISCLOSURES

AstraZeneca also provided the medications and a placebo inhaler for the study. Dr. S. Rennard and S. Siddiqui were paid employees of AstraZeneca at the time the project was designed and performed. Dr. R. Casaburi has been a consultant for AstraZeneca.

## AUTHOR CONTRIBUTIONS

W.W.S., J.P., H.B.R., S.S., S.R., and R.C. conceived and designed research; W.W.S., J.P., and M.C. performed experiments; W.W.S., J.P., M.C., and R.C. analyzed data; W.W.S., J.P., M.C., H.B.R., and R.C. interpreted results of experiments; W.W.S., J.P., and M.C. prepared figures; W.W.S. and J.P. drafted manuscript; W.W.S., J.P., H.B.R., S.R., and R.C. edited and revised manuscript; W.W.S., J.P., M.C., H.B.R., S.S., S.R., and R.C. approved final version of manuscript.
